# An Efficient and Robust Star Identification Algorithm Based on Neural Networks

**DOI:** 10.3390/s21227686

**Published:** 2021-11-19

**Authors:** Bendong Wang, Hao Wang, Zhonghe Jin

**Affiliations:** School of Aeronautics and Astronautics, Zhejiang University, Hangzhou 310013, China; wanggeck@zju.edu.cn (B.W.); jinzh@zju.edu.cn (Z.J.)

**Keywords:** star identification, modified log-polar mapping, one-dimensional Convolutional NeuralNetwork

## Abstract

A lost-in-space star identification algorithm based on a one-dimensional Convolutional Neural Network (1D CNN) is proposed. The lost-in-space star identification aims to identify stars observed with corresponding catalog stars when there is no prior attitude information. With the help of neural networks, the robustness and the speed of the star identification are improved greatly. In this paper, a modified log-Polar mapping is used to constructed rotation-invariant star patterns. Then a 1D CNN is utilized to classify the star patterns associated with guide stars. In the 1D CNN model, a global average pooling layer is used to replace fully-connected layers to reduce the number of parameters and the risk of overfitting. Experiments show that the proposed algorithm is highly robust to position noise, magnitude noise, and false stars. The identification accuracy is 98.1% with 5 pixels position noise, 97.4% with 5 false stars, and 97.7% with 0.5 Mv magnitude noise, respectively, which is significantly higher than the identification rate of the pyramid, optimized grid and modified log-polar algorithms. Moreover, the proposed algorithm guarantees a reliable star identification under dynamic conditions. The identification accuracy is 82.1% with angular velocity of 10 degrees per second. Furthermore, its identification time is as short as 32.7 miliseconds and the memory required is about 1920 kilobytes. The algorithm proposed is suitable for current embedded systems.

## 1. Introduction

Attitude information is required for most spacecraft missions, such as telecommunication, Earth observation, space exploration, celestial navigation, and so on. Star trackers are widely used for attitude determination because they provide more accurate attitude information than other attitude measurement devices. Generally, a star tracker takes an image of the celestial sky and uses the information in the image to identify the stars in the field-of-view(FOV). The attitude is then calculated by using the star vectors and a attitude determination algorithm.

The most critical part of a star tracker is star identification. Star identification algorithms fall into two categories: lost-in-space algorithms and recursive algorithms. The lost-in-space identification is more essential and difficult because no prior attitude information is available. Thus, achieving a reliable lost-in-space star identification algorithm has been a challenging problem in the past few decades.

The existing LIS star identification algorithms can be classified roughly into two categories: subgraph isomorphism based algorithms and pattern recognition based algorithms. The subgraph isomorphism based algorithms tends to approach star identification as an instance of subgraph isomorphism [1]. In this case, the angular distances between the stars are used to find the relevant isomorphic subgraph in a database. The most representative algorithm is the triangle algorithm [2], which is easy to implement, but three angular distances are not sufficient to avoid mismatches. Later, Mortari et al. [3,4] modified this algorithm to pyramid algorithm. Four or more stars are used in pyramid algorithm to match measured star patterns, so it has a high success rate when encountering false stars. However, this method requires more identification time with the increasing of the number of false stars. Besides the triangle-based algorithms, other methods such as the geometric voting algorithm were proposed in recent years [5]. The geometric voting algorithm uses a geometric voting scheme built on pairs of stars to guarantee robustness against positional noise and false stars. However, in the case of higher position noise or magnitude noise, its identification rate will decrease significantly. In order to deal with a large number of false objects, Schiattarella et al. [6] designed a multi-pole algorithm utilizing multiple verification procedures. This algorithm is robust to false stars and moderate angular velocities. Recently, Schiattarella et al. [7] improved the multi-pole algorithm and proposed the rolling shutter compensation method to deal with the false stars and high angular velocity.

Another kind of approaches are pattern based algorithms. Every star is associated with a pattern that describes the position of the star and its neighboring stars. The first pattern based algorithm is the grid algorithm [1]. The grid algorithm is more robust and faster than triangle algorithms. Many improvements were made to the grid algorithm to improve its performance [8,9]. These modified grid algorithms performs better than the original grid algorithm when encountering magnitude noise and false stars. However, the main defect of grid algorithm is that its recognition depends on the correct selection of the reference star’s closest neighboring star. Zhang et al. [10] proposed a radial and cyclic algorithm to avoid incorrect selection of the closest neighboring star by making patterns based on radial directions, but this approach is sensitive to magnitude noise. In 2019, Wei et al. [11] used the dynamic cyclic features to suppress the position noise and magnitude noise. Other feature extraction methods such as log-polar transform and singular value decomposition are used for star identification [12,13]. The algorithm basing on log-polar transform is relatively slow due to the computationally intensive string matching [14]. The modified algorithm based on log-polar transform is then proposed to reduce the time consumed and enhance the robustness of star identification [15], but similar to the grid algorithm, it needs to find the correct closest neighboring star. Thus, too many false stars may lead the misidentification. The advantage of the singular value decomposition is that the pattern recognition and the attitude estimation can be performed simultaneously, but the algorithm used the magnitude information of stars which may be affected by magnitude noise. More recently, Sun et al. [16] proposed a modified SVD algorithm that is independent of the star magnitude information, so it is more robust to magnitude noise, but its performance may be affected by false stars too. Kim proposed another modified SVD algorithm for use in dynamic scenarios [17], the method is robust to positional noise and false stars, but the robustness against false stars is not reported.

Neural networks were used in star identification as early as 1989 [18]. In 2000, Hong proposed a algorithm using a neural network and fuzzy logic to identify the stars [19]. Although this algorithm performs much faster than some traditional algorithms, the parallel hardware required is not available at that time. In 2012, Jiang proposed a method combining the triangle algorithm, grid algorithm and neural network [20]. This algorithm is more robustness to noise compared with the traditional triangle algorithm, but the influence of false stars was not taken into consideration. More recently, Xu proposed a representation learning based star identification network called RPNet [21]. Simulation results show that the algorithm is quite robust towards small deformations of the star image and star magnitude noise. However, too many false stars or missing stars will significantly decrease the identification rate. Rijlaarsdam proposed another neural network for star identification [22]. The method is robust to false stars and position noise, but the magnitude noise it considered is too small. In our previous work [23], VGG16 is used for star identification, the algorithm is robust to various noise, but the network requires large memory. Compared with conventional star identification algorithms, the neural network based approaches can achieve a time complexity of only O(1), but these neural networks contain fully-connected layers and require large amount of memory [14].

To solve problems mentioned above, a one-dimensional convolutional neural network based algorithm is developed in this paper. The proposed idea is to use modified log-Polar transform(LPT) to construct a star pattern from the star image, then a one-dimensional convolutional neural network (1D CNN) with global average pooling is applied to classify star patterns. Comparing with the pyramid, optimized grid and modified LPT algorithms, the proposed method is more robust to varies of noise. In addition, it requires less memory than other neural network based algorithms and can be implemented on current embedded systems.

The main contributions of this paper are:

1. A modified Log-Polar transform is used for star pattern construction. With the help of modified LPT, the training time of the network is reduced and the robustness of the network is improved.
2. A 1D CNN is introduced for star pattern classification. The designed network could deal with position noise, magnitude noise, false stars and angular velocities.
3. The global average pooling is introduced into the 1D CNN network to reduce the size of the network. Consequently, the designed network can be implemented on on-board processors.

The rest of this paper is organized as follows: In Section 2, we introduce the modified log-polar transform and the structure of the 1D CNN. In Section 3, the implementation and the performance of the proposed algorithm are denoted. In Section 4, conclusions and future work are presented.

## 2. Algorithm Description

In this section, a modified log-polar transform is derived. After that, the structure of the 1D CNN is described as well as the training dataset.

### 2.1. Star Pattern Construction

Deep convolutional neural networks are empirically known that they are invariant to moderate translation but sensitive to rotation in image classification, thus it is necessary to create a rotational invariant star pattern. Since rotation and scaling in the Cartesian coordinate system can be converted to translations in the polar coordinate system, the log-polar transform is applied to construct the star pattern [24,25]. The log-polar mapping of the star points from Cartesian coordinates $\left(x_{i},y_{i}\right)$ to log-polar coordinates $\left(\rho_{i},\theta_{i}\right)$ is defined as:

(1) ρi=log((xi−xc)2+(yi−yc)2)θi=arctan((yi−yc)(yi−yc)(xi−xc)(xi−xc))

Here, $\left(x_{c},y_{c}\right)$ is the coordinates of the reference star $S_{c}$. Then θ and ρ can be combined as input vectors. According to [26], the normalization, of the input data affects the performance of deep learning models. Also, stars that are closer to the reference star are more important than others, because they are less likely to be missing. Hence, it is good to use a modified log-polar transform to modify ρ to improve the performance of the network. A modified log-polar mapping is defined as follows:

(2) ρi=ξ−klog((xi−xc)2+(yi−yc)2)θi=arctan((yi−yc)(yi−yc)(xi−xc)(xi−xc))

Here, we introduce two constants ξ and *k* to modulate $\rho_{i}$. The training results will show that the modified LPT is more suitable to construct the input vector. Then the log-polar coordinates of star points are converted to 1-D vector $P_{s}$ by the equation:

(3) Ps(n)=ρin=θi(360∘360∘NsNs)

where $N_{s}$ is the length of $P_{s}$. If there is no neighbor star falling in the *n*-th angle interval, $P_{s}\left(n\right)$ will be set to zero. The input size of the network is 1×224, so if $N_{s}$ is smaller than 224, we concatenate two $P_{s}$ to form an 1×224 input vector $P_{in}$ as follows.

(4) $$\begin{array}{cc} \; & P_{in}\left(i\right)=P_{s}\left(i\right)\;i=1,2\ldots N_{s}\\ \; & P_{in}\left(i\right)=P_{s}\left(i-N_{s}\right)\;i=\left(N_{s}+1\right),\left(N_{s}+2\right)\ldots 224 \end{array}$$

### 2.2. Neural Network Architecture

**Figure 1:**
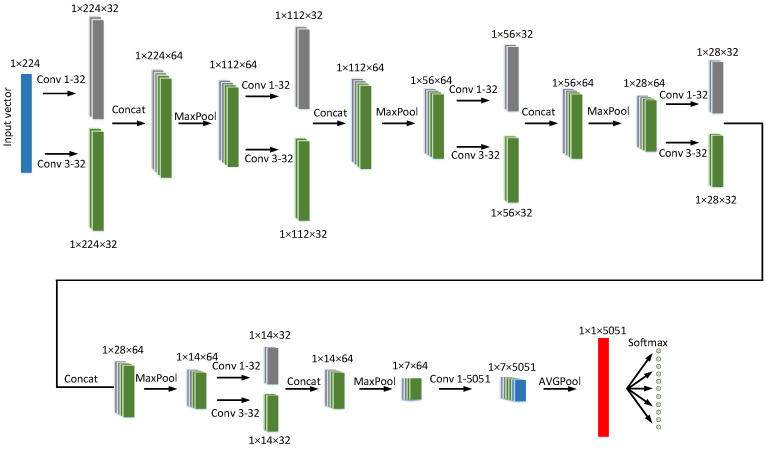
Structure of the Network.

As the star pattern is a 1×224 vector, the star identification problem is then converted to a sequence recognition. There are several kinds of neural network models can be applied to sequence recognition, such as Deep Convolutional Neural Network (DCNN), Recurrent neural networks (RNN) or Convolutional Recurrent Neural Network (CRNN) [27,28]. We designed a 1D-CNN, it’s architecture is shown in Figure 1.

A brief description of the structure is as follows:

CONV layer: After the input layer, multiple convolutional layers with 1×3 convolution kernels and 1×1 convolution kernels are used to produce the feature map. The convolutional layer parameters are denoted as “Conv<receptive field size>-<number of channels>”. For example, “Conv3-32” denotes a convolutional layer with 32 1 × 3 filters. The last CONV layer with 1×1 convolution kernel is used to increase the number of feature map to the number of guide stars.

Batch normalization: Batch normalization is performed after every CONV layer to enable higher learning rate so that the network convergence rate and the robustness will be improved.

Activation function: The activation function of each convolutional layer is the leaky rectified linear unit (ReLU) function. It can be represented as:

(5) $$f\left(x\right)=\left\{\begin{array}{c} x\;if\;x>0\\ \alpha x\;if\;x\leq 0 \end{array}\right.$$

where α is set to 0.05.

Concat layer: The Concat layer is used to concatenate multiple feature maps to one group. The concatenation is channel-wise.

Pooling layer: The max pooling is performed over 1×2 windows with stride 2 after each Concat layer.

Global average pooling: Fully connected layers require large memory due to the fully connectivity. Therefore, the global average pooling is adopted after several CONV and pooling layers. The global average pooling over the fully connected layer can reduce the number of parameters substantially, so the tendency of over fitting is reduced due to the elimination of parameters [29].

The softmax formula is as follows:

(6) $$p_{n}=\exp\left(z_{n}\right)/\sum_{k=1}^{M}\exp(z_{k})\;n\in \left[1,M\right]$$

where $p_{n}$ denotes the predicted probability of the *n*-th star, and *z* is the the array of the output neurons.

### 2.3. Construction of the Training Dataset

To obtain the network with better performance, a training dataset is constructed. The Smithsonian Astrophysical Observatory (SAO) star catalog is chosen as the basic star catalog. The highest visual magnitude detected by the star sensor under the static condition is set to 6.0 Mv. Guide stars should meet the following requirements:

1. The magnitude of the guide star should be less than 6.0 Mv.
2. Double stars or binary stars are labelled as a single star.

**Figure 2:**
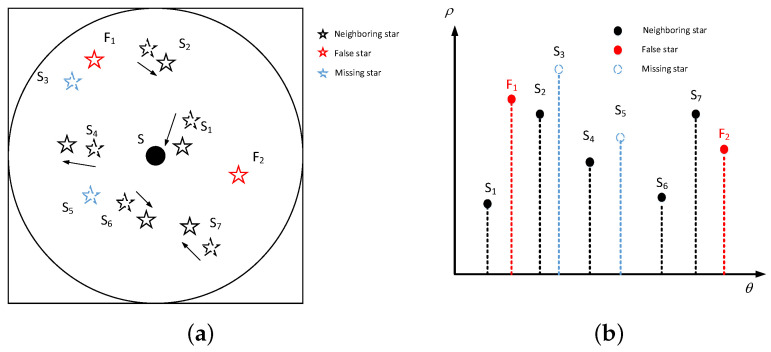
(**a**) original star image. (**b**) constructed star pattern.

Based on these rules, a guide star catalog containing 5051 stars is obtained. After that, the training dataset can be constructed as following:

Step1: Select a guide star as the reference star $S_{c}$. Set the optic axis of the star tracker to point at *S*, which means the projection of $S_{c}$ lies at the center of the image. Then the neighboring stars $S_{i}$ appearing in the field of view are also projected from celestial coordinate system into the image coordinate system as shown in Figure 2a.
Step2: The modified LPT transform is performed for every neighboring stars. A set of logarithmic distances and relative angles is obtained. Then same to the vector construction procedure, the log-polar coordinates of the neighboring stars are discreted and a 1×224 vector $P_{s}$ is constructed, as shown in Figure 2b. $P_{s}$ is considered as the basic pattern of the reference star $S_{c}$.
Step3: Data augmentation is performed to reduce overfitting and enhance the generalization power of the network, which is also the major way to enhance the robustness of the algorithm. The details of data augmentation are described as follows:

- Random position deviations varying from −5 pixels to 5 pixels were added to each star’s coordinates. As shown in Figure 2a, the curves with arrow denotes the direction of translation of each star.
- A random magnitude deviation varying from −0.5 Mv to 0.5 Mv were added to each star. Therefore, some stars might appear or disappear due to the magnitude noise. As shown in Figure 2, $S_{3}$ and $S_{5}$ are the missing stars, and the corresponding elements of $P_{s}$ is set as 0.
- One to five false stars with random positions and magnitudes are added. As shown in Figure 2, $F_{1}$ and $F_{2}$ are the false stars added in the scene.
- In order to improve the rotation invariance of the algorithm, the basic pattern $P_{s}$ is shifted from $0^{\circ }$ to $360^{\circ }$ by $1^{\circ }$ step.
- Dynamic condition would decrease the magnitude limit of the star tracker and lead to missing stars. In order to make the network robust to dynamic condition, the magnitude limit is set from 4 Mv to 6 Mv, and the stars with a magnitude beyond the limit will be dropped.

For each guide star in catalog, we generated 1000 training samples according to the above steps.

### 2.4. Star Identification Algorithm

**Figure 3:**
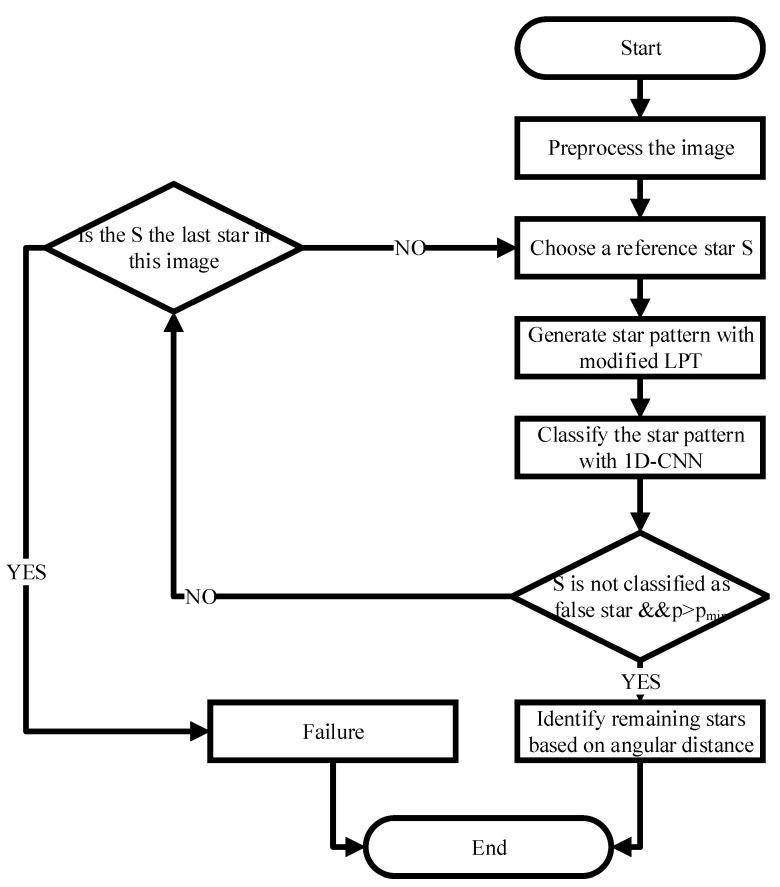
Flowchart of the star identification algorithm.

As shown in the Figure 3, the basic flow of the algorithm is as follows:

1. Image Preprocessing. Star points are extracted via centroid extraction algorithm.
2. Reference Star Determination. The nearest star to the center of the image is taken as the reference star *S*.
3. Star Pattern Construction. The star pattern is generated by modified LPT.
4. Star Pattern Classification. Input the star pattern to the proposed network, then the ID of the reference star and the corresponding probability ρ are obtained.
5. Validation. Unless the reference star is not classified as a false star and $\rho >\rho_{min}$, this identification is considered as a success. Otherwise, the reference star may be a false star or the ID is wrong. Then a new reference star will be selected, which means to go back to step 2.
6. Remaining Stars Identification. Identify the remaining stars in the image by the angular distances between them and the reference star.

## 3. Experiments and Results

### 3.1. Training of the Network

**Figure 4:**
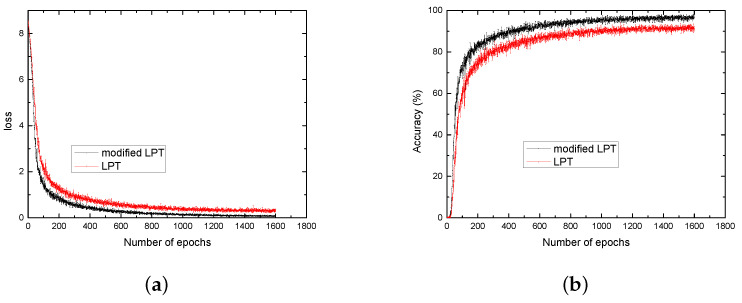
(**a**) Loss vs. the number of epochs. (**b**) accuracy vs. the number of epochs.

The network was trained with the Caffe framework [30]. The modified log-polar transform and log-polar transform were used to construct training datasets, respectively. In simulation, the parameters of the modified log-polar transform ξ and *k* were set as 12 and 1.5, respectively. The stochastic gradient descent (SGD) method with a batch size of 8 examples was employed for the training. The base learning rate was set to 0.01 and learning rate decay policy was set to step with a step size of 10,000. Momentum and weight decay were set to 0.9 and 0.0005, respectively. The plots of loss/accuracy using the two training datasets are shown in Figure 4. As shown, the modified log-polar transform improved the accuracy and the training speed of the network. When the number of the epochs is 1600, the loss of the network with modified log-polar transform is about 0.1, while the loss of the network with log-polar transform is still over 0.25. The accuracy of the network with modified log-polar transform is about 4% higher than the network with log-polar transform.

### 3.2. Comparison and Analysis

The proposed algorithm were verified and compared with other studies on a series of simulated star images and real images. The parameters of the optical system used in simulation are shown in Table 1. The images were simulated according to [31,32]. The discretization factor $N_{s}$ was set as 180. The performance of the proposed algorithm is compared with those of the optimized grid [9], pyramid [3] and modified LPT algorithms [15]. These algorithms are evaluated under different noise conditions, i.e., position deviation, false stars and magnitude uncertainty. The influence of the dynamic condition is also presented. Note that the pyramid algorithm is more suitable for identifying a measured star pattern with four or more stars. Thus, images containing at least four stars are tested.

#### 3.2.1. Robustness to Star Positional Noise

**Figure 5:**
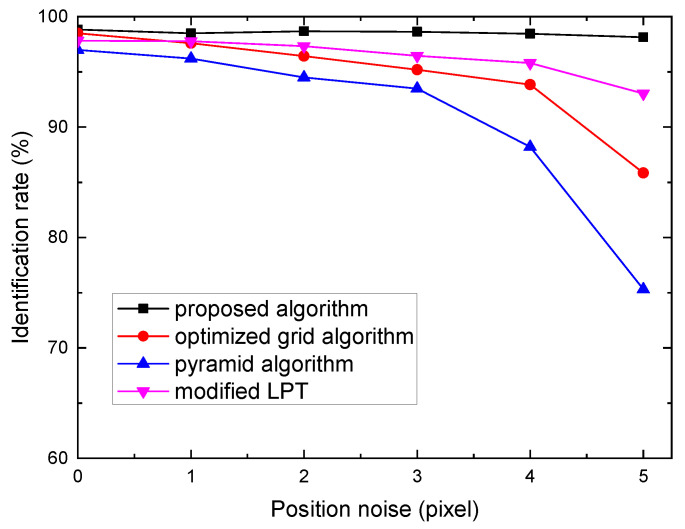
Identification rate versus position deviation.

The positional noise is usually caused by thermal deformations or vibrations of the optical system, motion of star tracker, the star centroid algorithm, detector imperfections, etc. In simulations, each star’s coordinates were added a position noise, which is a random Gaussian noise with a zero mean and a certain variance. The standard deviation was set from 0 to 5 pixels with a increment of 1 pixels. The performances of the four algorithms are shown in Figure 5. As shown in Figure 5, the identification rate of proposed algorithm is superior over other algorithms. When the positional noise is 5 pixels, the identification rate of the optimized grid algorithm, pyramid algorithm, modified LPT algorithm and the proposed algorithm are 85.8%, 75.3%, 93.1%, 98.1%, respectively. Obviously, the pyramid algorithm is more sensitive to the position noise, because the position noise affects the geometric distance between stars and that causes a redundant identification or misidentification. The optimized grid algorithm and modified LPT algorithm are robust to small position noise, but large position noise may change the grid pattern or LPT pattern and causes a misidentification.

#### 3.2.2. Robustness to False Stars

**Figure 6:**
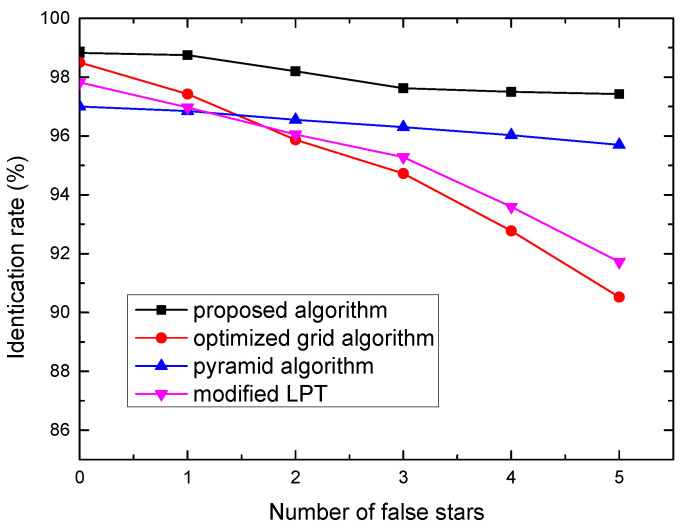
Identification rate versus number of false stars.

There are various factors that may lead to false stars, such as planets, space debris, single Event Upsets, sensor aging, and thermal drift. The influence of false stars on the performance of the four algorithms is shown in Figure 6. The number of false stars increased from 0 to 5, and each with a random magnitude and a random position. Clearly, the proposed algorithm and pyramid algorithm performs better than other algorithms, their identification rates remain higher than 95% in the case of 5 false stars. Meanwhile, the optimized grid algorithm and modified LPT algorithm’s identification rates decrease from 98.5% to 90.5% and 97.8% to 91.7%. False stars may be selected as the closest neighboring stars and impact grid pattern or LPT pattern, thus when there are too many false stars presenting in the star image, the performance of the optimized grid algorithm and modified LPT algorithm will degrade.

#### 3.2.3. Robustness to Magnitude Noise

**Figure 7:**
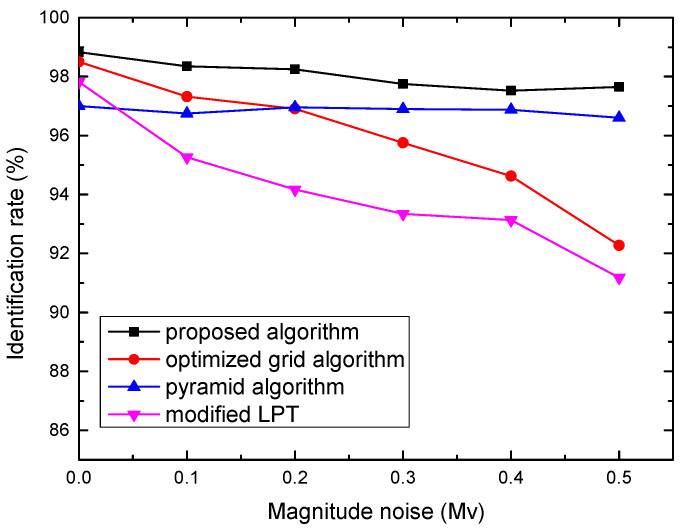
Identification rate versus magnitude noise.

The stellar instrument magnitude is predicted with the spectral response of the star tracker and the spectral characteristics of the star. The computation error of the stellar instrument magnitude may lead to missing stars or false stars. To simulate such a scenario, we applied a magnitude noise to each star’s magnitude. The standard deviation of the magnitude noise was set from 0 to 0.5 Mv with an increment of 0.1 during simulation. The maximum magnitude deviation was assigned to 0.5 Mv for each star. Figure 7 shows the performance of these algorithms. It shows that magnitude noise has little influence on the proposed algorithm and pyramid algorithm. When the magnitude noise is 0.5 Mv, the identification rates of the proposed algorithm and pyramid algorithm are 97.7% and 96.6%, respectively. In contrast, the rate of the optimized grid algorithm and modified LPT algorithm drops quickly from 98.7% to 92.3%, and from 97.6% to 91.1%, respectively.

#### 3.2.4. Robustness to Rotation Velocity of the Star Tracker

When star tracker works under dynamic condition, the rotation of the star tracker will cause the star image to elongate during exposure time, causing a star streak. This will decrease the signal to noise ratio (SNR) and lead to missing stars and missing stars. Assuming the angular velocity is constant during the exposure time. According to [33], The path of star centroid on the image is given by:

(7) $$\left[\begin{array}{c} \overset{•}{x}\\ \overset{•}{y} \end{array}\right]=\left[\begin{array}{c} \begin{array}{ccc} \frac{xy}{f_{\gamma }} & -(f_{\gamma }+\frac{y^{2}}{f_{\gamma }}) & y \end{array}\\ \begin{array}{ccc} \left(f_{\gamma }+\frac{y^{2}}{f_{\gamma }}\right) & -\frac{xy}{f_{\gamma }} & x \end{array} \end{array}\right]\left[\begin{array}{c} \omega_{x}\\ \omega_{y}\\ \omega_{z} \end{array}\right]$$

where $f_{\gamma }$ denotes the ratio of focal length to pixel size; $\omega_{x}$, $\omega_{y}$ and $\omega_{z}$ represent the angular rate about three axis. In our simulation, the angular velocity was set from $1^{\circ }/s$ to $10^{\circ }/s$ with an increment of $1^{\circ }/s$, and the direction of the rotation axis was set randomly.

**Figure 8:**
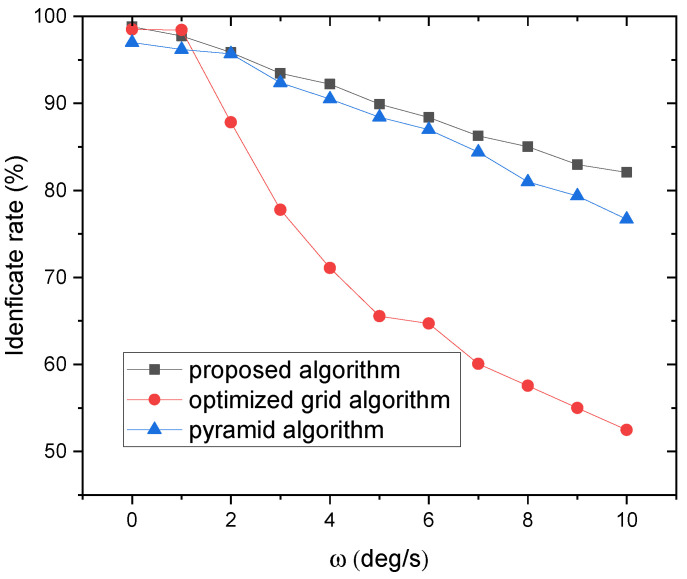
The effect of the rotation on the magnitude limit for star detection.

The experimental results are shown in Figure 8. As can be seen, the identification rate of the proposed algorithm, optimized grid algorithm and pyramid algorithm decreases from 97.7% to 82.1%,98.5% to 52.46% and 97% to 76.7% as the rotation velocity increases from $1^{\circ }/s$ to $10^{\circ }/s$. The reason for the decline of the identification rate is that the angular rate causes too many stars missing.

#### 3.2.5. Performance of the Proposed Idea on Real Images

**Figure 9:**
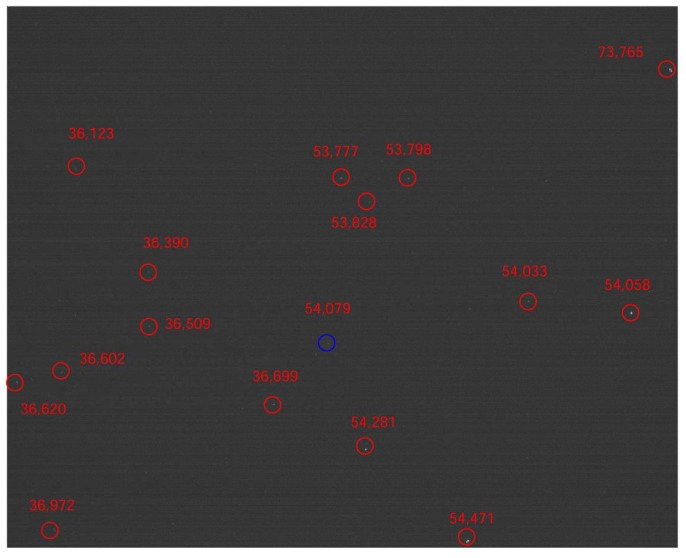
The real star image.

The algorithm was also tested on real star images. The proposed technique was able to identify 1458 images correctly out of the 1472 images, achieving an identification accuracy of 99.05%. In Figure 9, star 54,079 is chosen as the reference star, the identified neighboring stars are marked in the image.

#### 3.2.6. Time and Memory Performance

The identification time and memory consumption are the key indicator for evaluating the performance of star identification algorithms. The proposed algorithm was first test on a NVIDIA Tegra X2 platform. The average identification time is 17 ms. But the NVIDIA Tegra X2 is not suitable for star trackers. To evaluate the time performance for the proposed algorithm better, we implemented the proposed algorithm and other algorithms on a TMS320C6747 DSP with the CPU frequency of 300 MHz. A 256 Mb SDRAM MT48LC64M4A2 was used as the external memory device of the DSP. TI’s C674x DSPLIB was used to optimize the code.

The identification time and memory consumption of the four algorithms on computer are listed in Table 2. As shown, the identification time of the proposed algorithm is 32.7 ms, which is much shorter than other three algorithms. This is because the input pattern is a sparse vector, all the zero elements can be skipped from convolution or multiplication to improve the computational time. Also the C674x DSPLIB provides several optimized DSP routines for matrix operations, which significantly shortened the identification time.

The memory requirement of the proposed algorithm is 1920.6 KB, which is larger than the optimized grid and modified LPT algorithms, but smaller than the pyramid algorithm. Since the size of the SDRAM is 256 Mb, storing 2 Mb parameters is not a question for this platform.

## 4. Conclusions

A lost-in-space identification algorithm combines a modified log-polar transform and 1D-CNN is proposed in this paper. The algorithm uses a modified log-polar transform to construct one-dimensional star patterns, which can improve the training speed and accuracy of the network. Then a 1D-CNN is designed to classify star patterns and a training dataset is constructed accordingly. With the global average pooling technique, the size of proposed network is reduced. Experiments show that this algorithm is highly reliable despite the star position noise, magnitude noise, false stars and angular velocity. Moreover, the algorithm was implemented on a DSP platform. The results demonstrate that the algorithm is efficient and the memory size is acceptable in application. Future work will focus on improving robustness under high dynamic conditions and the hardware performance.

## Figures and Tables

**Table 1:** Parameters for simulations.

Parameter	Value
**Resolution of CMOS**	2048×2048
**Size of One Pixel**	5.5μm×5.5μm
**FOV**	$14.5^{\circ }\times 14.5^{\circ }$
**Highest Visual Magnitude**	6.0 Mv
**Full Well Charge**	13,500$e^{-}$
**Temporal Noise**	13$e^{-}$
**Dark current signal**	125$e^{-}/s$
**Fixed Pattern Noise**	<1 LSB
**Photon non-uniformity response noise**	<1% RMS of signal
**Astronomical Background**	10 Mv
**Exposure time**	16 ms

**Table 2:** The identification time and memory consumption of the three algorithms.

Algorithm	Identification Time	Memory Consumption
**Proposed algorithm**	32.7 ms	1920.6 KB
**Pyramid algorithm**	341.2 ms	2282.3 KB
**Optimized Grid algorithm**	178.7 ms	348.1 KB
**Modified LPT algorithm**	65.4 ms	313.5 KB

## Data Availability

Not applicable.
